# *Leishmania* spp.-Infected Dogs Have Circulating Anti-Skeletal Muscle Autoantibodies Recognizing SERCA1

**DOI:** 10.3390/pathogens10040463

**Published:** 2021-04-12

**Authors:** Francesco Prisco, Davide De Biase, Giuseppe Piegari, Francesco Oriente, Ilaria Cimmino, Valeria De Pasquale, Michele Costanzo, Pasquale Santoro, Manuela Gizzarelli, Serenella Papparella, Orlando Paciello

**Affiliations:** 1 Department of Veterinary Medicine and Animal Productions, University of Naples Federico II, 80137 Naples, Italy; davide.debiase@unina.it (D.D.B.); giuseppe.piegari@unina.it (G.P.); valeria.depasquale@unina.it (V.D.P.); manuela.gizzarelli@unina.it (M.G.); papparel@unina.it (S.P.); paciello@unina.it (O.P.); 2 Research Unit (URT) Genomic of Diabetes, Department of Translational Medicine, Institute of Experimental Endocrinology and Oncology, National Council of Research (CNR), University of Naples Federico II, 80131 Naples, Italy; foriente@unina.it (F.O.); ilaria.cimmino@unina.it (I.C.); 3 Department of Molecular Medicine and Medical Biotechnology, Medical School, University of Naples Federico II, 80131 Naples, Italy; michele.costanzo@unina.it; 4 CEINGE—Biotecnologie Avanzate s.c.ar.l., 80145 Naples, Italy; 5 Veterinary Diagnostic Laboratory (Di.Lab.), 80125 Naples, Italy; pasantor@unina.it

**Keywords:** animal model, canine, muscle, myositis, protozoa, leishmaniasis, infectious, autoimmunity, antigen mimicry, IgG

## Abstract

*Leishmania* spp. infection is associated with an inflammatory myopathy (IM) in dogs. The pathomechanism underlying this disorder is still elusive, however, the pattern of cellular infiltration and MHC I and II upregulation indicate an immune-mediated myositis. This study aimed to investigate the presence of autoantibodies targeting the skeletal muscle in sera of leishmania-infected dogs and individuate the major autoantigen. We tested sera from 35 leishmania-infected dogs and sera from 10 negative controls for the presence of circulating autoantibodies with indirect immunofluorescence. Immunoblot and mass spectrometry were used to identify the main target autoantigen. Immunocolocalization and immunoblot on immunoprecipitated muscle proteins were performed to confirm the individuated major autoantigen. We identified circulating autoantibodies that recognize skeletal muscle antigen(s) in sera of leishmania-infected dogs. The major antigen was identified as the sarcoplasmic/endoplasmic reticulum Ca^2+^-ATPase 1 (SERCA1). We also found that canine SERCA1 presents several identical traits to the calcium-translocating P-type ATPase of *Leishmania infantum*. In the present study, we defined circulating anti-SERCA1 autoantibodies as part of the pathogenesis of the leishmania-associated IM in dogs. Based on our data, we hypothesize that antigen mimicry is the mechanism underlying the production of these autoantibodies in leishmania-infected dogs.

## 1. Introduction

Inflammatory myopathies (IMs) are a large and heterogeneous group of acquired disorders characterized by inflammatory cells directly responsible for initiating and maintaining myofiber injury [1,2,3]. The majority of these disorders have an unknown cause and are defined as idiopathic IMs (IIMs), while others have been associated with exposure to infectious and non-infectious agents and are defined as secondary IMs [2,4,5]. Information about IM in veterinary medicine is marginal and is based on biological plausibility from animal models and human medicine [6].

In humans, IMs are subclassified as dermatomyositis (DM), polymyositis (PM), inclusion body myositis (IBM) and immune-mediated necrotizing myopathy (IMNM) [2,3]. In dogs, the most common IMs include the highly specialized immune-mediated masticatory muscle myositis (MMM) and PM, with extraocular myositis and DM occurring less commonly [7]. IMs homologous to human IBM and IMNM are not yet well reported in dogs but a few case reports of canine myopathies that share many features with these IM subtypes have been published [7,8].

The etiology and the pathomechanisms underlying IMs are still largely unknown. The majority of these disorders are considered autoimmune disorders in which skeletal muscle is inappropriately targeted by the immune system [2,7,9]. Although the disease mechanisms for IMs are ill-defined, the innate immune system (including cytokines and chemokines) and adaptive immune system (including autoantibodies and antigen-specific T cells) are probably involved. Furthermore, several non-immune-mediated mechanisms contribute to IM pathogenesis, including cell stress pathways, free radicals, altered energy metabolism, protein homeostasis and mitochondrial damage [2,10].

Several genetic risk factors have been described in humans [2], but no genetic risk variants have been identified in other animals to date. However, the overrepresentation of some canine breeds (e.g., Boxer and Newfoundland) in relatively large clinicopathological studies allows us to hypothesize that the genetic variants play a role at least in this species [11,12]. Moreover, mounting evidence suggests that several environmental risk factors are involved in the pathogenesis of IMs [2]. Among them, both infectious and non-infectious factors are reported [2]. IMs have been related to different spontaneous infections in humans [2], dogs [13], cats [14] and horses [15], and a relation has also been proposed in sheep [10]. Notably, *Leishmania infantum* infection has been related to a form of IM in dogs, which has been experimentally reproduced in Syrian hamsters and mice [13,16,17,18].

Around 70 animal species, including humans, have been found to be natural reservoir hosts of *Leishmania* parasites [19], however, infected dogs constitute the main domestic reservoir of the parasite and play a key role in transmission to humans [20]. *Leishmania infantum* (syn. *L. chagasi*) has been identified as the main etiologic agent of canine leishmaniasis. However, other Leishmania species (e.g., *L. donovani*, *L. braziliensis*, *L. tropica*, *L. major,* etc.) are able to infect and induce disease both in dogs and in other animals [20,21,22,23].

The pathogenesis of *Leishmania* infection in dogs is extremely complex and is the result of the interaction among the vector (e.g., repeated infectious bites), parasite (virulence) and host (e.g., genetic background, immune response, coexisting diseases) [22]. The wide variability of these factors results in a wide range of clinicopathological presentations ranging in severity from self-healing cutaneous leishmaniasis (CL) to fatal disseminated visceral leishmaniasis (VL) leading to organ damage and dysfunction [21,22]. Other than IM, the clinicopathological picture of *Leishmania infantum*-infected dogs may include various combinations of exfoliative and/or ulcerative dermatitis, with or without nasodigital hyperkeratosis and onychogryphosis, glomerulonephritis, myocarditis, anterior uveitis, keratoconjunctivitis sicca, epistaxis and/or polyarthritis [13,16,22]. Although the pathogenetic mechanisms underlying these features are not yet clear, the best characterized mechanisms are the presence of circulating immune complexes [24] and the production of autoantibodies directed against different structures of the host, including anti-nuclear [25], anti-platelet [26] and anti-smooth muscle autoantibodies [27].

Leishmania-related IM can be classified as a canine PM [5,6]. Myopathic features related to Leishmania infection are necrosis, regeneration, fibrosis and infiltration of lymphocytes (mainly CD8+) and macrophages. As observed in other PMs [10,15,18,28], Leishmania-related IM is also further characterized by a wide sarcolemmal Major Histocompatibility Complex (MHC) class I and II overexpression [13]. Moreover, an interesting myopathic observation is the presence of non-necrotic MHC I-positive myofibers invaded by CD8-positive lymphocytes (CD8/MHC I complex) that strongly suggest an immune-mediated pathogenesis [13,28,29]. Leishmania infection in dogs is also related to myocarditis that shares many histological characteristics with Leishmania-related myopathy [16]. Furthermore, MHC I and II overexpression on the cardiomyocyte membrane is also reported [16]. Due to the numerous shared pathological features between Leishmania-related myopathy and cardiomyopathy and due to many similarities between these two tissues, a common immune-mediated pathogenetic mechanism has been postulated [6,16].

Based on our observation and the literature [2,5,13,16,17,18], we hypothesize that one of the main components of the pathogenesis of canine Leishmania-related myositis is the dysregulation of the adaptive immune system with the production of autoantibodies directed against muscle structures.

In the present study, we have recognized circulating autoantibodies against skeletal muscle in Leishmania-infected dogs and identified the main target antigen, which is a member of the sarcoplasmic reticulum calcium ATPases located on the sarcoplasmic reticulum and named sarcoplasmic/endoplasmic reticulum Ca^2+^-ATPase 1 (SERCA1).

## 2. Results

### 2.1. Leishmania-Infected Dogs Have Circulating Autoantibodies Recognizing Skeletal Muscle

To investigate the presence of circulating autoantibodies of anti-skeletal muscle in Leishmania-infected dogs, we analyzed 35 sera with anti-Leishmania antibody titers ranging from 1/80 to 1/1280 and 10 negative control sera using indirect immunofluorescence (IF) on sections of normal dog muscle. To reduce the background signal, the sera were pooled and purified. To prevent fluorescein isothiocyanate (FITC)-conjugated antibodies bound to the endogenous dog IgG, present in blood vessels and the interstitial spaces, we pretreated normal dog muscle sections with F(ab’)_2_ fragments of rabbit anti-dog IgG before incubation of the sections with serial dilutions of test serum. 

**Figure 1:**
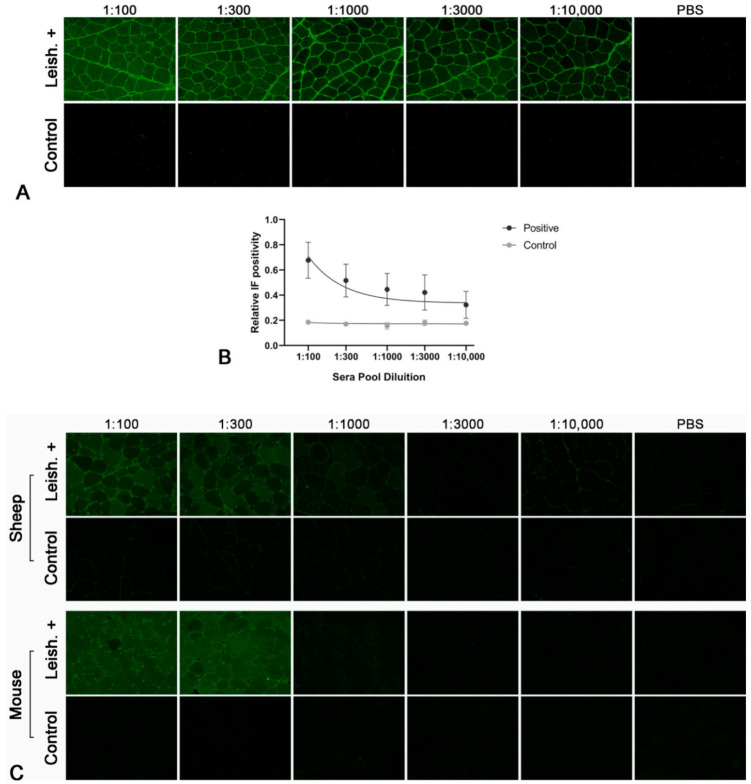
Indirect immunofluorescence (IF) using dog, sheep and mouse muscle and different dilutions of pooled and purified sera from Leishmania-infected dogs (Leish.+) and normal control dogs (Control). (**A**) Sera pool from Leishmania-infected dogs showed antibodies against skeletal muscle in titers up to over 1:10,000. At 1:100 and 1:300 dilutions, there are no evident differences in the staining of the sarcoplasm of the different muscle fibers; on the other hand, starting from the 1:1000 sera dilution, a differential stain among fibers was evident with a checkerboard pattern. Sera pool from control dogs did not show positive staining. (**B**) Scatter plot with error bars of the quantitative assessment of IF positivity on canine muscle sections. The X-axis represents sera dilutions (logarithmic scale) and Y-axis represents relative IF positivity (linear scale). The IF positivity of pooled sera from Leishmania-infected dogs is dilution dependent (r_s_ = 0.662896; *p* = 0.000014). In contrast, this association is not evident in the control sera (r_s_ = 0.169621; *p* = 0.598178). (**C**) For both sheep and mouse muscle, myofiber staining with a checkerboard pattern was evident until the 1:300 dilution of the pooled and purified sera from Leishmania-infected dogs. Sera pool from control dogs did not show positive staining.

All pooled sera from Leishmania-infected dogs showed antibodies against skeletal muscle in titers up to over 1:10,000 (Figure 1A). The IF showed mainly a sarcoplasmic positivity. At higher concentrations, the sarcoplasm of all fibers was positive; on the other hand, usually starting from the 1:1000 sera dilution, a differential stain among fibers was evident with a checkerboard pattern. None of the control sera pools had detectable antibodies in this test.

Fisher’s exact test confirmed a statistically significant difference between Leishmania-positive and negative pools (*p* = 0.027778). The IF positivity was directly correlated with the dilution of pooled sera from Leishmania-infected dogs (r_s_ = 0.662896; *p* = 0.000014). In contrast, this correlation was not evident in the control sera (r_s_ = 0.169621; *p* = 0.598178; Figure 1B).

### 2.2. Species Specificity of the Autoantibodies

We also performed the indirect IF assay on sections of normal sheep and mouse muscle to determine if the autoantibodies found in the Leishmania-infected dogs are specific to canine muscle. Positivity was demonstrated both in sheep and in mouse muscle with all pooled sera from Leishmania-infected dogs. The IF positivity showed a sarcoplasmic checkerboard pattern that was evident from a 1:100 dilution. The intensity of the staining was lower on sheep muscle and mouse muscle compared with dogs. No staining was found using pooled sera from normal control dogs on muscle from both species (Figure 1C).

### 2.3. Antibodies in Leishmania-Infected Dogs Recognize a Specific Muscle Protein

**Figure 2:**
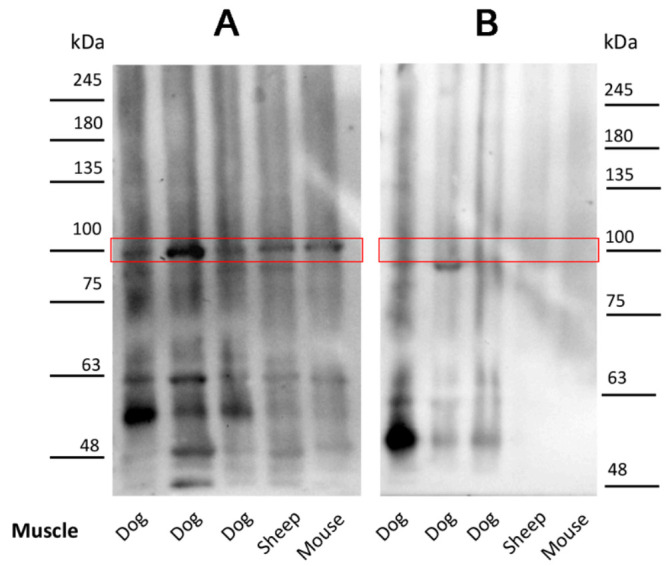
Identification of major antigens of the circulating anti-skeletal muscle autoantibodies. Autoantibodies in sera from Leishmania-infected dogs (**A**) bind to a protein of about 100 kDa. This protein band was not detected in the control dog sera (**B**). The muscle samples were named according to the species: dog, sheep and mouse.

We performed an immunoblot analysis using the sera from the Leishmania-infected dogs on normal muscle protein extract to identify the unknown antigen(s). A band corresponding to about 100 kDa was identified in muscle samples immunoblotted with the Leishmania-positive sera (Figure 2A). This protein band was not detected in the muscle extracts when the sera of normal control dogs were used for the immunoblot analysis (Figure 2B). Additionally, proteins with a molecular mass lower than and greater than 100 kDa were identified in the immunoblotting assay; however, these proteins were not consistently identified by all sera.

### 2.4. Isolation and Characterization of the Canine Muscle Protein Recognized by the Leishmania-Infected Dog Sera

**Figure 3:**
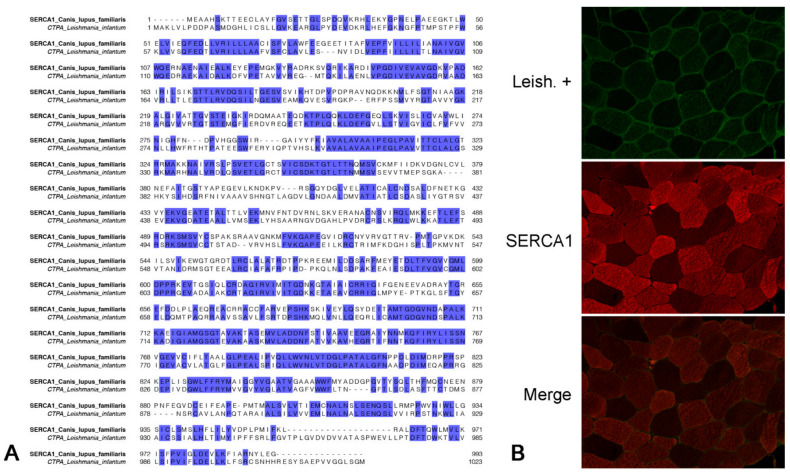
Protein sequence alignment and fluorescent immunocolocalization. (**A**) Canine sarcoplasmic/endoplasmic reticulum calcium ATPase 1 (SERCA1, UniProt ID: E2RRB2) is 49.02% identical to calcium-translocating P-type ATPase (CTPA, UniProt ID: A4HRZ6) of *Leishmania infantum*. Identical amino acids are highlighted with blue. (**B**) Double-color immunofluorescence with purified sera pool of Leishmania-infected dogs (a: green, fluorescein isothiocyanate, FITC) and commercial mouse anti-SERCA1 antibodies (b: red, tetramethylrhodamine, TRITC) and their colocalization (c: merge, orange) on normal dog skeletal muscle sections. A marked immunocolocalization between SERCA1 and the major antigen is evident.

To identify the target protein of the canine autoantibodies, the protein extracts from normal canine muscles were fractionated by SDS-PAGE and the resulting bands with a molecular weight of about 100 kDa were excised, trypsinized and analyzed by liquid chromatography–tandem mass spectrometry (LC–MS/MS). LC–MS/MS analysis led to the identification of sarcoplasmic/endoplasmic reticulum calcium ATPase 1 (SERCA1) (UniProt ID: E2RRB2, gene name: ATP2A1) in the muscle extracts of the analyzed dogs. The confident identification of SERCA1 occurred with more than two peptides in all the MS runs. Thus, the canine SERCA1 was subjected to basic local alignment search tool (BLAST) analysis, showing that this protein is highly conserved across species, including humans and other primates, cats, ferrets, mice, horses, pigs, bovines, sheep, etc. Particularly, canine SERCA1 amino acid sequences shared 96.18% and 95.77% identity with murine (NP_031530.2) and ovine (XP_004020912.1) SERCA1, respectively. The BLAST search also revealed that the canine SERCA1 has significant overall homology (49.02%) with calcium-translocating P-type ATPase of *Leishmania infantum* (XP_001462838.2) and that these proteins share many identical traits (Figure 3A).

### 2.5. Muscle Protein Recognized by Antibodies of Leishmania-Infected Dogs Colocalizes with Anti-SERCA1 Antibodies

To evaluate the colocalization of the muscle protein recognized by the antibodies of Leishmania-infected dogs and SERCA1, we performed a double indirect immunofluorescence on sections of fresh frozen normal canine muscle. Muscle sections were incubated with commercial anti-SERCA1 antibodies and pooled and purified sera of Leishmania-infected dogs. Anti-SERCA1 antibodies marked type II muscle fibers with a checkerboard pattern, and the staining partially colocalized with the muscle antigens recognized by the circulating antibodies of Leishmania-infected dogs (Figure 3B).

### 2.6. Antibodies of Leishmania-Infected Dogs Recognize an Immunoprecipitated SERCA1 Protein

**Figure 4:**
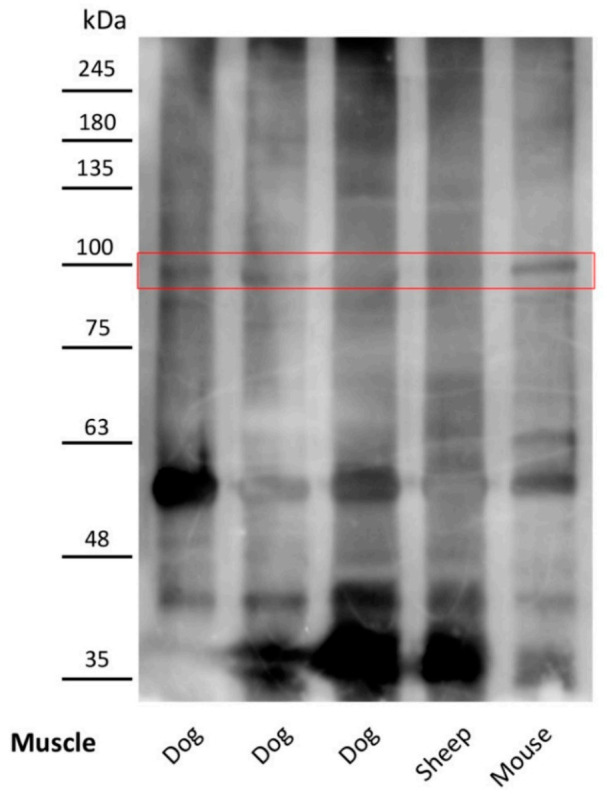
Immunoblot using total muscle protein samples immunoprecipitated with anti-SERCA1 antibody and sera of Leishmania-infected dogs. Muscle tissues were homogenized and solubilized. Total protein samples were immunoprecipitated with anti-SERCA1 antibody and immunoblotted with the sera of Leishmania-infected dogs. The sera from Leishmania-infected dogs bind to a protein consistent with SERCA1. The autoradiograph shown is representative of three different experiments. The muscle samples were named according to the species: dog, sheep and mouse.

SERCA1 was isolated through immunoprecipitation from normal muscle protein extract using a commercial anti-SERCA1 monoclonal antibody. The immunoprecipitate was tested by immunoblotting with the Leishmania-infected dog sera. A protein band consistent with SERCA1 was identified in the muscle samples immunoblotted with sera from the Leishmania-infected dogs (Figure 4). SERCA1 was not detected in the muscle extracts using the sera of control dogs.

## 3. Discussion

*Leishmania spp*. infection has been associated with an IM and a myocarditis in dogs [13,16,30]. A morphological characterization of these disorders is available [13,16], however, the underling pathomechanisms are still elusive [13,16]. An autoimmune mechanism has been hypothesized [13,16]; therefore, the aim of this study was to investigate the presence of circulating autoantibodies recognizing skeletal muscle in Leishmania-infected dogs.

In this study, we identified circulating IgG autoantibodies recognizing skeletal muscle proteins in Leishmania-infected dogs. We found mainly a sarcoplasmic positivity with indirect IF and we also identified SERCA1 as the main target antigen.

Circulating immune complexes and several autoantibodies have already been described in sera of Leishmania-infected dogs [31]. In a study of 44 infected adult dogs, antinuclear antibodies have been reported in up to 30% of dogs with an indirect IF method [32]. It has also been established that these antibodies are often directed against DNA-associated proteins, such as histones, and that are part of the pathogenetic mechanism of Leishmania-associated glomerulonephritis [25]. In a large study of 260 dogs, anti-actin and anti-tubulin IgG has been reported, respectively, in 95% and 94% of dogs infected with *Leishmania donovani* using ELISA [31]. Furthermore, anti-mammalian basal membrane glycoproteins and cerebroside antibodies have been described [31]. Circulating anti-skeletal muscle autoantibodies have also been found in other infection-related IMs, such as piroplasmosis in horses [15] and feline immunodeficiency virus infection in cats [33]. 

In the present study, we consistently found autoantibodies directed against SERCA1 in Leishmania-infected dogs. Sarcoplasmic–endoplasmic reticulum calcium ATPase (SERCA) is responsible for transporting calcium (Ca^2+^) from the cytosol into the lumen of the sarcoplasmic reticulum (SR) following muscular contraction in both cardiac and skeletal muscle [34]. SERCAs are transmembrane proteins with three major isoforms and several sub-isoforms: SERCA1 isoforms are expressed in the fast-twitch (type II) skeletal muscle fibers. SERCA2a is expressed in cardiomyocytes, slow-twitch skeletal muscle fibers (type I) and vascular smooth muscle cells. SERCA2b is expressed ubiquitously and SERCA2c has recently been reported to be expressed in the left ventricle in humans, while SERCA3 proteins can be expressed in various tissues including hematopoietic cell lineages [34,35].

The selective expression of SERCA1 in type II skeletal muscle fibers explains the observed checkerboard pattern with IF in our study. In canine muscle, this pattern was not evident at low sera dilutions, suggesting a cross-reaction of the described autoantibodies with SERCA2 isoforms (expressed by type I fibers) which can appear at low sera dilutions or in the presence of other anti-skeletal muscle autoantibodies at lower concentrations.

Since we found positivity on sheep and mouse muscles, we assume that autoantibodies are directed to phylogenetically preserved muscle antigens. These data are also supported by the reported high homology between sheep and mouse SERCA1 evaluated by in silico analysis. The lower antibody titers against muscle from other species may indicate the existence of multiple antigens, some of which are species specific, or suggest that the affinity of the antibodies for the antigen(s) in other species is lower [7]. The above hypotheses are not mutually exclusive, and they do not exclude the formulation of other hypotheses.

In our study, SERCA1 was found to be the main antigen, most frequently recognized by the sera tested. However, proteins with a molecular mass less than and greater than 100 kDa were identified in immunoblotting with the tested dog sera. The significance of autoantibodies to these other antigens is not known, because they were not found in all cases. Future investigations are needed to understand the pathogenetic significance of these other presumed autoantibodies. A plurality of autoantibodies is reported in different IMs, including canine masticatory muscle myositis [36,37]. Therefore, different autoantigens are also expected in Leishmania-associated IM. Autoantibodies in IM are generally classified in myositis-specific autoantibodies (MSAs) or myositis-associated autoantibodies (MAAs), depending on their prevalence in other related conditions [37]. The identification of MSAs in Leishmania-associated myositis and myocarditis is important because they could be used as biomarkers helping the diagnosis, prognosis and monitoring of these diseases [37]. Muscle biopsy will remain the gold standard for diagnosing myositis; however, the evaluation of this biomarker(s) would also be a useful tool for clinicians, helping in the decision to perform a muscle biopsy.

In our study, the lack of information about the clinical symptoms of the dogs can be considered a limitation to be solved with further studies focused on the correlation between muscle pathology and circulating autoantibodies in canine leishmaniasis. A second limitation of the present study is represented by the need to pool the sera and to purify them to reduce the background signal in IF. This allowed us to identify the major antigen, however, it prevented us from performing an accurate assessment of the prevalence of these autoantibodies in Leishmania-infected dogs. The methods used in the present study were necessary to morphologically identify the location and precisely identify the target antigens [15,36], however, to adequately study the prevalence of these autoantibodies in Leishmania-infected dogs, it is appropriate to perform other studies testing individual canine sera. Moreover, using different methods, including WB and ELISA, can be useful to identify the one with the highest sensitivity. 

The role of autoantibodies in causing muscle damage and dysfunction is debated because most of the autoantigens are intracellular and thus not easily accessible to circulating autoantibodies [37,38]. SERCA proteins, being expressed on the sarcoplasmic reticulum, are indirectly in contact with the extracellular milieu through the T-tubular system [39]. Among the various autoantibodies described during IM and myocarditis, autoantibodies to SERCA2a have been detected in humans with myocarditis or dilated cardiomyopathy [35,40]. A model of experimental myocarditis has been generated by immunizing mice with SERCA2a [35,40]. This model allowed to define, through immunoperoxidase staining and transmission electron microscopy, that anti-SERCA2a antibodies gain access through the transverse tubules of the myocardium that are connected to the interstitial extracellular environment [39]. Considering the shared ultrastructural features between skeletal and cardiac muscle, we hypothesize that the same mechanism can explain how circulating autoantibodies can bind SERCA1.

SERCA1 is expressed in canine skeletal muscle [41], thus, the presence of autoantibodies directed to SERCA1 may explain the reported Leishmania-related myositis [13]. On the other hand, the expression of SERCA1 in cardiomyocytes in dogs is less well characterized. Some evidence of mRNA expression in mammalian cardiomyocytes has been reported [42] and the abnormal protein expression of SERCA1 in cardiomyocytes has been related to dilated cardiomyopathy [41], suggesting that, at least in this condition, the myocardium may be the target of anti-SERCA1 autoantibodies. Furthermore, we can hypothesize that the described anti-SERCA1 autoantibodies partially cross-react with the other SERCA isoforms, expressed in the myocardium, or that Leishmania-infected dogs have other autoantibodies recognizing proteins shared by skeletal and cardiac muscle.

Different hypotheses have been formulated to explain the production of autoantibodies during infections [13,16]. One of the most solid is antigen mimicry. It is hypothesized that the proteins of the infectious agent have epitopes in common with host proteins, therefore, the autoantibodies produced against these epitopes cross-react with the host proteins, causing an autoimmune pathology [13,16]. This mechanism has been well established in human toxoplasmosis [43]. In humans, during *Trypanosoma cruzi* infection, circulating autoantibodies against β_1_-adrenergic receptor were detected. These autoantibodies determine an immune-mediated myocarditis [43]. The production of such antibodies was explained by molecular mimicry between the immunodominant ribosomal protein PO of *Trypanosoma cruzi* and a functional epitope on the human β_1_-adrenergic receptor [43].

We hypothesize that antigen mimicry may explain the production of autoantibodies against SERCA1. This hypothesis is based on the high homology between protein calcium-translocating P-type ATPase of *Leishmania infantum* and canine SERCA1 and the identification of perfectly overlapping traits of amino acid sequence between these two proteins. Our results are not sufficient to prove this mechanism, however, they strongly support our hypothesis. Further studies are needed to clarify the pathogenetic mechanisms underlying the production of anti-SERCA1 antibodies in Leishmania-infected dogs.

## 4. Materials and Methods

### 4.1. Sera

Thirty-five Leishmania-positive dog sera and 10 negative controls were selected from the serum bank of the Department of Veterinary Medicine of University Federico II in Napoli and the Veterinary Diagnostic Laboratory (Di.Lab.), Naples. All sera were tested for anti-leishmanial antibody titers using an immunofluorescent antibody test (IFAT) for *Leishmania* spp. Serum data are summarized in Table 1. No clinical information of the dogs was recorded at the time of serum selection. Upon arrival, the sera were stored at −80 °C until further processing.

To reduce background signals in IF and unspecific signals in immunoblots, groups of sera from 5 dogs were randomly pooled in 7 pools of Leishmania-positive dogs and 2 groups of negative controls. IgGs were purified from the pooled sera using the Protein A IgG Purification Kit (Thermo Fisher Scientific, Waltham, MA, USA) according to the manufacturer’s instructions.

### 4.2. Tissues

To perform the immunofluorescence assays and to obtain the muscle protein extract for the immunoblots and immunoprecipitation, frozen samples from the quadriceps femoris of heathy dogs, mice and sheep were retrospectively selected from the tissue archives of the Comparative Neuromuscular Laboratory of the Department of Veterinary Medicine of University Federico II in Napoli. Each sample was frozen in isopentane precooled in liquid nitrogen and stored at −80 °C until further processing [44]. All selected muscles (Table 2) were collected from animals serologically and parasitologically negative for *L. infantum.* Muscle changes were excluded with our routine panel of stains for muscle biopsies [29], including hematoxylin and eosin (HE) and Engel trichrome (ET) for a basic morphologic evaluation and mitochondria distribution; reduced nicotinamide adenine dinucleotide–tetrazolium reductase (NADH-TR) to observe the intermyofibrillar pattern and secondary distribution of mitochondria; and succinate dehydrogenase (SDH) and cytochrome oxidase (COX) to evaluate the activity and distribution of mitochondria.

### 4.3. Indirect Immunofluorescent Staining

Eight-micrometer transversal cryosections were cut from selected muscle specimens and dried at room temperature for 45 min. Sections were fixed in acetone for 10 min at 4 °C. After 3 washes of 5 min in PBS, sections were incubated with 10% normal rabbit serum in PBS for 30 min at room temperature. To block endogenous dog IgG, after 3 washes of 5 min in PBS, sections of dog muscles were preincubated with F(ab’)_2_ fragments of rabbit anti-dog IgG (H + L) (1:50; Rockland Immunochemicals, Limerick, PA, USA) for 1 h. The latter step was avoided for the muscles of the other species. Serum pools from dogs were serially diluted in PBS (1:100, 1:300, 1:1000, 1:3000, 1:10,000) and, after 3 washes of 5 min in PBS, were added to each section for incubation overnight at 4 °C. Control sections were incubated with PBS. After washing three times with PBS for 5 min, FITC-conjugated rabbit anti-dog IgG (H + L) (1:300; Jackson Laboratories, West Grove, PA, USA) was added to each section and incubated for 40 min at room temperature. Sections were washed three times with PBS for 5 min and mounted under coverslips in VECTASHIELD^®^ H-1100 (Vector, Burlingame, CA, USA) to prevent fading of fluorescence [15].

A quantitative assessment of immunofluorescence-stained sections was performed for each dilution for each serum pool [45,46]. Ten 40 × fields were randomly photographed under an optical microscope (Leica DM6000B by Leica, Wetzlar, Germany) coupled with a digital camera (Leica DFC450C digital camera by Leica). The intensity of the fluorescence signal was measured for each photo with Fiji (ImageJ, National Institutes of Health). The mean stain intensity of the 10 analyzed fields was calculated for each dilution for each serum pool.

### 4.4. Indirect Immunofluorescent with Colocalization

To evaluate the colocalization of detected autoantigens and SERCA1, cryosections were processed as described in the section on indirect immunofluorescent staining up to the primary antibody step. As a first primary antibody, purified serum pool 1 from Leishmania-infected dogs diluted 1:1000 overnight at 4 °C was used. After washing three times with PBS for 5 min, FITC-conjugated rabbit anti-dog IgG (H + L) (1:300; Jackson Laboratories, West Grove, PA, USA) was added to each section and incubated for 40 min at room temperature. After the slides were rinsed with PBS, a second primary mouse monoclonal antibody directed against SERCA1 ATPase (1:200; clone VE121G9, Thermo Fisher Scientific, Waltham, MA, USA) was applied for 2 h at room temperature. A TRITC fluorochrome-labeled rabbit anti-mouse secondary antibody (1:300; Jackson Laboratories, West Grove, PA, USA) was applied on sections for 1 h at room temperature. Slides were rinsed with PBS and mounted with VECTASHIELD® H-1100 (Vector, Burlingame, CA, USA) [10]. 

### 4.5. Western Blot Analysis and Immunoprecipitation Procedures

Tissue samples were homogenized in a Polytron (Brinkmann Instruments, Westbury, NY, USA) in 1 ml T-PER reagent/100 mg of tissue according to the manufacturer’s instructions (Pierce, Rockford, IL, USA). The homogenate was stirred for 2 h at 4 °C and then centrifuged at 14,000 rpm × 20 min. The supernatant was collected, and proteins were determined by the Bradford procedure [47]. Proteins from total homogenates were separated by SDS-PAGE. Aliquots of the lysates were precipitated with SERCA1 ATPase antibodies coupled to protein G Sepharose for 2 h at 4 °C. Immunoprecipitation and Western blot analysis were performed as previously described [47,48].

### 4.6. LC–MS/MS Analysis for Antigen Identification

Protein extracts obtained from dog muscle tissues were fractionated by SDS-PAGE. The gel was stained using the Gel Code Blue Stain Reagent (Thermo Fisher Scientific, Waltham, MA, USA) and a band approximately near to the 100 kDa molecular weight marker was excised from the gel lanes and subjected to in-gel digestion, as reported elsewhere [49]. Trypsin (Promega, Madison, WI, USA) was used as a proteolytic enzyme [49]. Then, peptide mixtures were analyzed by LC–MS/MS on an LTQ-Orbitrap XL (Thermo Scientific, Bremen, Germany) mass spectrometer equipped with a nanoLC system (nanoEasy II), as already reported [50,51]. Briefly, samples were injected onto a 2 cm trapping column (C18, ID 100 μm, 5 μm) and fractionated using a 20 cm C18 reverse-phase silica capillary column (ID 75 μm, 5 μm). A non-linear gradient of 60 min was used at a flow rate of 250 nL/min to elute peptides. MS analysis was performed using data-dependent acquisition (DDA) at an m/z range from 200 to 1800 Da, followed by fragmentation of the five most intense doubly, triply and quadruply charged ions. Protein identification was carried out using Mascot 2.4 (Matrix Science, Boston, MA, USA) through the MS/MS Ions Search tool. The *Canis lupus familiaris* database was selected as the taxonomy.

### 4.7. Statistical Analysis

Fisher’s exact test was used to compare the difference of the frequency of positivity to the IF assay between Leishmania-positive and -negative pools. Spearman’s rank correlation coefficient was used to evaluate the correlation between serum dilution and positivity to the IF assay.

## 5. Conclusions

This study provides evidence that Leishmania-infected dogs have circulating IgG autoantibodies recognizing different skeletal muscle protein, among which SERCA1 is the major recognized antigen. Our results also strongly suggest that the production of these autoantibodies can derive from a molecular mimicry mechanism between calcium-translocating P-type ATPase of *Leishmania infantum* and canine SERCA1.

Finally, further research is needed to explore the diagnostic utility and accuracy of anti-SERCA1 autoantibodies in IM and myocarditis in Leishmania-infected dogs. The identification of MSAs in Leishmania-associated IM and myocarditis are of interest because they are useful biomarkers in helping the diagnosis, prognosis and monitoring of these diseases.

## Figures and Tables

**Table 1:** Serum samples with corresponding anti-leishmanial antibody titers tested by immunofluorescent antibody test (IFAT).

Serum #	IFAT
1, 2	1/80
3–8	1/160
9–12	1/320
13–15	1/640
16–35	1/1280
36–45	Absent

**Table 2:** Muscle samples with corresponding species, breed or strain, sex and age.

Muscle #	Species	Breed/Strain	Sex	Age
1	Dog	Siberian husky	M	5 years
2	Dog	Mix	M	12 years
3	Dog	Jack Russell terrier	M	9 years
4	Dog	Whippet	F	6 years
5	Dog	Mix	M	3.5 months
6	Mouse	C57	F	1 year
7	Mouse	C57	F	1 year
8	Mouse	C57	F	1 year
9	Sheep	Mix	F	4 years
10	Sheep	Mix	F	5 years
11	Sheep	Mix	F	7 years

## Data Availability

The data presented in this study are available on request from the corresponding author.
